# Molecular Pathology and Personalized Medicine: The Dawn of a New Era in Companion Diagnostics—*Practical Considerations about Companion Diagnostics for Non-Small-Cell-Lung-Cancer*

**DOI:** 10.3390/jpm6010003

**Published:** 2016-01-15

**Authors:** Till Plönes, Walburga Engel-Riedel, Erich Stoelben, Christina Limmroth, Oliver Schildgen, Verena Schildgen

**Affiliations:** 1 Lungclinic Merheim, Department of Thoracic Surgery, Lung Clinic Cologne, Kliniken der Stadt Köln gGmbH, Cologne Merheim Hospital, Faculty of Health/School of Medicine, Witten/Herdecke, Ostmerheimerstrasse 200, 51109 Köln, Germany; ploenest@kliniken-koeln.de (T.P.); engelriedelq@kliniken-koeln.de (W.E.-R.); stoelbene@kliniken-koeln.de (E.S.); 2 Clinics for Internal Medicine Holweide, Hospital of Cologne, Neufelder Str. 34, 51067 Köln, Germany; limmrothc@kliniken-koeln.de; 3 Institute for Pathology, Hospital of Cologne, Private University Witten/Herdecke, Ostmerheimerstrasse 200, 51109 Köln, Germany

**Keywords:** companion diagnostics, lung cancer, ALK, FISH, EGFR, KRAS

## Abstract

Companion diagnostics (CDx) have become a major tool in molecular pathology and assist in therapy decisions in an increasing number of various cancers. Particularly, the developments in lung cancer have been most impressing in the last decade and consequently lung cancer mutation testing and molecular profiling has become a major business of diagnostic laboratories. However, it has become difficult to decide which biomarkers are currently relevant for therapy decisions, as many of the new biomarkers are not yet approved as therapy targets, remain in the status of clinical studies, or still have not left the experimental phase. The current review is focussed on those markers that do have current therapy implications, practical implications arising from the respective companion diagnostics, and thus is focused on daily practice.

## 1. Introduction

**Figure 1:**
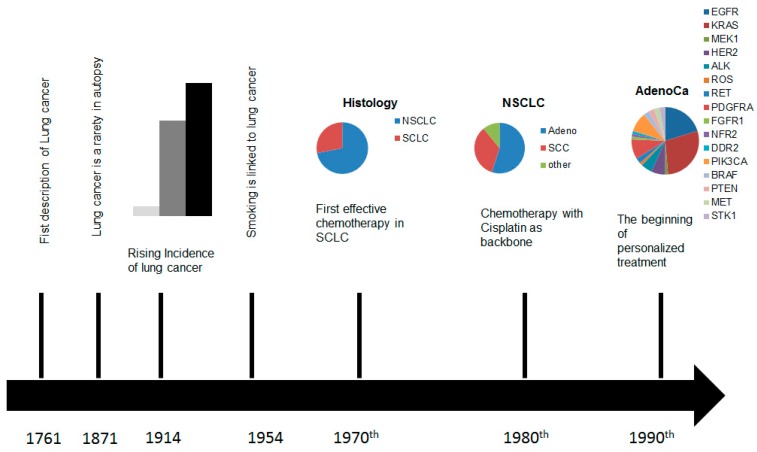
Time scale of the developments in the diagnostics and treatment of lung cancer.

The treatment of non-small cell lung cancer (NSCLC) has dramatically changed over the last decades. Since the wide spread of array technology and next generation sequencing, several driver mutations, including gene rearrangements, have been identified, leading to a better understanding of many oncological targets and the development of new small-molecule drugs, intervening in pathways, which control growth, differentiation, and metastasis of lung cancer cells [1,2,3,4,5,6]. These findings changed the view of the oncological community on lung cancer. For the first decades of the twentieth century lung cancer was considered as a homogenous disease and a small number of patients with local tumour load were treated surgically by pneumonectomy, and later on by lobectomy. For the majority of patients with advanced disease no effective treatment was available [7]. In the 1970s lung cancer was divided into non-small cell lung cancer and small cell lung cancer (SCLC) and the first successful chemotherapies, especially in SCLC were established. In the 1980s, after screening a broad spectrum of cytotoxic agents, it became apparent that cisplatin as a backbone, combined with a second drug, improves survival and became the first-line standard of care in advanced small cell and non-small cell lung cancer [8] (Figure 1). During the following years, various immunohistochemical markers enabled pathologists to define more distinct histopathological subgroups, different therapeutic agents were developed, and their use in distinct histopathologic entities were optimized [2]. All this interdisciplinary research led to a median survival of 12, or more, months in patients with metastasising lung cancer [9,10,11,12,13]. The discovery of single mutations which could be used for cancer treatment in the beginning of the new millennium facilitated an individualized treatment even if mainly limited to non-smokers and adenocarcinomas (ACs). At the same time we learned to characterize lung carcinomas by radiological, morphological, immunohistological, and molecular changes [14]. These breakthroughs offered new treatment options and made the pathologist and the radiologist an integral part of a multidisciplinary team in lung cancer treatment.

## 2. Markers of Current Diagnostic and Therapeutic Relevance

Companion diagnostics should be intended to assist the team of pathologists, oncologists, and surgeons to decide if a therapy option for a specific tumour entity is likely to be successful or may fail due to natural resistance of the respective cancer types. Therefore, those assays must specifically identify a valid biomarker that can be used to determine between therapy-eligible and non-eligible patients. In addition to economic considerations, this procedure is also beneficial for the patient as it avoids the side effects of non-effective therapies and can help manage side effects of useful treatments.

In principle, any companion diagnostics assay has to fulfil four major criteria. (a) The assay needs to be specific; (b) should be sensitive and allow the prediction of a therapy outcome; (c) must be reproducible; and (d) should be easy to interpret [15,16]. The latter prerequisite is especially difficult to reach, as the mechanisms leading to cancer are complex and, thus, experts in molecular biology are required to perform and analyze those assays.

As a matter of discussion, it is frequently claimed that companion diagnostic assays require an FDA approval. Although this may be true due to national legal regulations in the United States of America and some studies in which US companies are involved, the FDA approval is not required in Europe and many other countries, nor is it a sign of quality for a specific assay [17]. Thus, it should be possible to choose those assay(s) that deliver the optimal test result and have the most benefit for the patient. In any case, CDx need to be properly validated with respect to their specificity, sensitivity, positive and negative predictive values, and reproducibility. Thereby, the validations need to include several ethnic groups as, e.g., a Caucasian wild-type may be considered a mutation in Asian patients, or as several allelic variants could occur in parallel [18,19,20].

### 2.1. Epidermal Growth Factor Receptor

The *epidermal growth factor receptor* (*EGFR*), also known as *HER-1* or *Erb1*, belongs to the *ErbB* receptor tyrosine kinase (RTK) family, which also includes other members like *HER-2/neu* (*ErbB2*), *HER-3* (*ErbB3*), and *HER-4* (*ErbB4*). These RTKs are mainly activated in two different ways: on the one hand, through the binding of specific ligands to the external receptor domain or through self-activation. Therefore, overexpression and increased release of ligands by tumour cells themselves or by cells of the tumor environment, like tumor-associated fibroblasts or macrophages, can result in a stimulation of the respective RTK [21]. On the other hand, the ligand-independent self-activation of RTKs results from different genetic mechanisms, leading to a permanent activation of the downstream pathways. First, amplification of the RTK gene results in an overexpression of the RTK [21,22,23]. Secondly, mutation of the RTK gene results in an altered RTK function. Finally, gene rearrangements, like translocations, can result in an ongoing activation or inactivation of the regulatory molecules [22,23].

**Figure 2:**
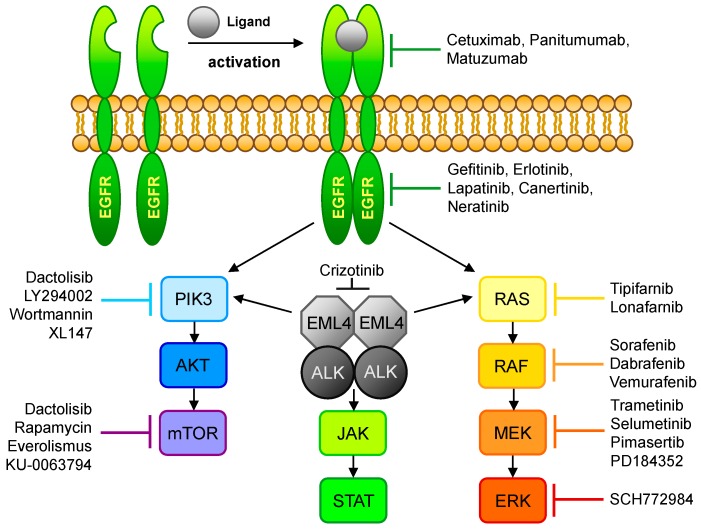
Overview of the *EGFR*- and *ALK*-signaling pathways and their respective inhibitors. Ligand binding to *EGFR* leads to dimerization of the receptor, resulting in phosphorylation of the tyrosine kinase domain and activation of the *PIK3*/*AKT*/*mTOR* or the *RAS/RAF/MEK/ERK* signaling pathway. This signal transduction cascade could also be activated via *EML4*-*Alk* which, in turn, may initiate the *JAK*/*STAT* pathway. The affected pathways control cell growth and play a role in cell differentiation.

A permanent activation of the *EGFR* TK affects signaling in the *RAS*-*RAF*-*MAP*, as well as in the *PIK3-AKT-mTOR*, pathway associated with proliferation, invasion, metastatic spread, and tumor angiogenesis (Figure 2). That therapy-relevant mutations appear in some NSCLC patients was already shown in 2004 by different groups [24,25,26,27,28,29,30]. Today, activating mutations of the *EGFR* gene are well-established molecular targets, but these first reports of responsiveness to tyrosine kinase inhibitors (TKIs) initiate a new kind of lung cancer treatment. Out of the four activating mutation hot-spots in the *EGFR* tyrosine kinase domain (exons 18–21), exon 19 and exon 21 respond best to TKI therapy, whereas exons 18 and 20 are less responsive [31]. In addition to these known hot-spots, a number of other *EGFR* mutations responding to TKI may exist, even if not described yet.

Altogether, about 15% of primary lung AC harbor *EGFR* mutations. Interestingly, *EGFR* gene mutations seem to occur more frequently in Asian patients than in Caucasians (30% *vs.* 8%) [32,33]. In the Caucasian population a mutation of the *EGFR* gene is more frequently found in AC with an acinar or papillary pattern (up to 27%), whereas the mucinous subtype is usually negative and often shows *KRAS* mutation instead. Mutation of the *EGFR* gene occur also more often in never-smokers than in ever-smokers (66% *vs.* 22%) and more often in Asiatic women than in Asiatic men (59% *vs.* 26%) [33,34]. Several clinical trials demonstrated that patients with *EGFR* mutations benefit from treatment with TKIs compared with standard of care chemotherapy measured as improved progression-free survival (PFS) and overall survival (OS) [35]. Today three *EGFR* inhibitors, afatinib (Giotrif), eroltinib (Tarceva), and gefitinib (Iressa), are FDA-approved. Due to the lower toxicity and the improved OS, current guidelines recommend TKIs for *EGFR* mutated patients as first-line therapy [36]. Additionally, TKIs can be offered as a treatment option for *EGFR* mutated patients with lower performance status [36].

It is important to note that despite the tumor entity, *EGFR* mutation could occur that leads to a resistance against TKIs, such as the mutation T790M that, in turn, is responsible for 50% of all *EGFR*-resistances against TKI [37,38].

Moreover it was shown that patients harboring an activating mutation in the *KRAS* gene or in the *BRAF* gene display a diminished response to TKI therapy with Erlotinib and Gefitinib; thus, it should be recommended to test treatment-naïve patients primarily for *KRAS* mutation which, in turn, are more frequent than EGFR mutations [27,39,40,41,42].

Methodically, *EGFR* is testing mainly performed by PCR or sequencing. Thereby, Sanger sequencing is replaced more and more by pyrosequencing that detects minor populations of mutated tumor cells more sensitively than Sanger sequencing [19,20,43]. In addition, qPCR methods have also been established as a widespread tool for the detection of known driver mutations [44,45,46,47,48], and for the detection of novel mutations and mutation patterns next generation sequencing is becoming the method of choice, provided the economical burden of this technology decreases as rapidly as the methodology is improved [48,49,50,51,52,53,54,55]. In this context it is to be expected that the percentage of cases with multiple mutations will increase.

A novel and promising approach for monitoring *EGFR*-TKI therapies could be the PCR analysis of liquid biopsies [56] that appear to identify major mutations in a large portion of patients, although the sensitivity and specificity of this technology requires improvement.

### 2.2. Anaplastic Lymphoma Kinase

With an incidence of 68 new cases per 100,000 people per year, an estimated total number of 350,000 new non-small cell lung cancer (NSCLC) cases are diagnosed each year in the European Union. Up to 10% of NSCLC patients are eligible for therapy with novel *ALK* (anaplastic lymphoma kinase) inhibitors as they have been diagnosed with a translocation in the gene coding for *ALK*. The *ALK* inhibitor therapy costs add up to approx. 9000 € per patient per month with treatment durations of up to one year. A recent study has shown that up to 10% of *ALK* cases are misdiagnosed by nearly 40% of pathologic investigations [57]. The current state-of-the-art *ALK* treatment comprises a fluorescent *in situ* hybridization (FISH) assay as diagnostic procedure accompanied by a therapy with the *ALK* inhibitor Crizotinib. Although detection of *ALK*-positive tumors via immunohistochemistry (IHC) can also be considered, the fluorescence *in situ* hybridization (FISH) represents the FDA-approved gold-standard for the detection of *ALK* rearrangements up to now. The therapy success ranges between a full therapy failure, and the complete remission of the tumor and the biomedical and systemic reasons for this range remain unknown so far, but it appears that the variety of different *ALK* mutations and variants contribute to the discrepant therapy results, especially as the state-of-the-art *Vysis* test only detects the *ALK* translocation, but not the respective partner. Although the major known fusion partner for *ALK* in NSCLC is the *echinoderm microtubule-associated protein-like 4* (EML4), of which 15 variants have been described, an additional number of 20 further *ALK* fusions with other genes are known, of which three have already been found in NSCLC [58,59,60].

In 2007, anaplastic lymphoma kinase (*ALK*) translocations were the second subset of oncogenic driver mutations discovered in NSCLC [61]. Mostly rearranged with *echinoderm microtubule-associated protein-like 4-AL-Kinase 1* (*EML4-ALK1*), but also with other oncogenic fusion partners, ALK signaling is activated by fusion with an upstream partner [62,63,64]. In physiological settings ALK is a transmembrane tyrosine-kinase receptor expressed mostly in the small intestine, testes, and the brain, but not in the lung. Rearranged with an oncogenic fusion partner, *ALK* is found in 2% to 7% of all NSCLC patients, which are usually younger never-smokers suffering from AC with a solid pattern or signet-ring cells [65]. In rare cases combination with other mutations like *EGFR* has been described [43,62,66,67,68,69]. Nevertheless, tumors harboring *ALK* translocations are resistant to *EGFR*-specific TKIs like erlotinib or gefitinib [27,30,70,71,72,73,74,75,76]. Instead, the FDA-approved ALK inhibitor Crizotinib demonstrated a superior performance in a phase III trial for second-line treatment of *ALK*-positive NSCLC.

The FDA approval of Crizotinib (Xalkori by Pfizer) in 2011 for the therapy of non-small cell lung cancer with translocations in the *anaplastic lymphoma kinase* (*ALK*) gene and the concurrently-approved companion diagnostic test (Vysis *ALK* Break-Apart FISH Probe by Abbott) opened the market for other companion diagnostic, FISH-based assays. Currently, it is crucial and required that molecular testing has to be performed before the personalized treatment with *ALK* inhibitors can be initiated, thus approx. 10%–50% of all NSCLC patients must receive *ALK* testing during their course of disease, resulting in 35,000 to 175,000 tests per year in Europe.

Unfortunately, the current FISH diagnostic assays do not produce clear “black or white” results but instead frequently give scope for the interpretation of assay results, leading to an unacceptable high error rate with the consequence of false therapies, especially as current studies show that the available assays do not detect all known variants of *ALK* fusions occurring in NSCLC [58,59,60].

In addition to the existence of various *ALK* translocations, the test principle itself is quite error-prone and highly dependent on optimal samples and experienced laboratory/medical staff. It is recommended that the test result is negative if less than five out of 50 counted tumor cells do not have the rearrangement, and is positive if 25 or more out of 50 counted tumor cells are rearranged. If 6–24 positive cells are counted, a second person should count another 50 cells, both results are added together, and then if 15% or more carry the rearrangement the sample is tested positive. This means that in the case of 15–24 counted cells the test would be positive even if the second analyst would count zero positive cells [77].

Furthermore it must be considered that none of the available FISH assays, except the one of Zytovision, delivers any information about the fusion partner in *ALK* rearrangement [64,78,79,80,81,82] and that, up to now, there is no information available if all variants of *ALK* fusions respond to the TKI therapy in the same way, leading to the conclusion that the therapy success of Crizotinib could depend on the ALK variant present in the respective tumor.

In cell cultures differential sensitivity to crizotinib was shown for some fusion variants, whilst it is yet unknown which are really biologically active in cancer patients and how the respective variants affect the response to Crizotinib treatment [64,78,79,80,81,82]. Moreover it has been demonstrated that translocated ALK genes can also be observed in healthy tissues and are not compulsorily associated with tumors [64,78,79,80,81,82]. Thereby, it is important to keep in mind that even for the known variants of *ALK* fusion genes the biological function is not known for all variants and that no information is available on the homogeneity of ALK fusion variants within a single tumor. Thus, an optimized *ALK* assay based on multiplex tools and combined with a sophisticated results interpretation system is highly desirable. However, any such new assay would require proper validation and for routine usage needs to pass national approvals (such as FDA) in order to become a full CDx rather than being a research-use-only product.

The long term impact of an optimized *ALK* assay will be a better patient stratification, optimized prognosis of therapy outcomes, improved clinical decisions, and safe healthcare budgets by reducing costs for false therapies.

A novel approach with promising results regarding congruence of the clinical outcome and the molecular diagnostics is a novel *ALK* antibody that detects the expressed *ALK* domain independent of the fusion partner [47]. However, if a rearrangement would lead to a frameshift mutation or to a non-expressed *ALK* variant the antibody would not identify the tumor as “positive”, although the sample could be positive by FISH and, thus, be eligible for Crizotinib therapy, and *vice versa*.

Finally, it is worth noting that the recommendation of how to use the FISH companion diagnostics is controversially discussed. The first German ring trial [83] recommended counting the split signal as positive signals if they were divided by one or more diameters of a signal, although it is the recommendation by the CDx manufacturers to count cells as positive if the split signals were divided by two or more signal diameters; consequently, the failure rate in the first trial was high and comparable to the rate of failure of the reference lab in which three of eight labs (37.5%) did not reach the internal thresholds [83]. This controversial discussion came up maybe exclusively in Germany, as the ring trial organizers recommended to count signals as positive if one diameter or more was between the split signals, whereas the FDA approved assay claims that two or more diameters are required for positive signals. Consequently, one can work conforming to the FDA, or conforming to the ring-trial, but in any case one would violate the FDA approved protocol or the national recommendation [57].

Regarding the currently-used methods it is worth noting that all methods have advantages and disadvantages. While FISH is able to detect *ALK* rearrangements on a molecular level, the interpretation of FISH analyses is sometimes difficult and requires experience of the pathologist or biologist who performs the analyses, and moreover it misses those cases in which *ALK* overexpression is dependent on other factors, such as promotor mutations [84]. IHC staining with *ALK* specific antibodies in contrast identifies those cases with an *ALK* expression in which Crizotinib is likely to be active, but, unfortunately, patients with an *ALK* mutation that is not expressed, but would be visible by FISH, are not identified, although they would be eligible for Crizotinib therapy by definition in the approval of the drug (if the drugs would be effective in these patients remains to be analyzed and discussed) [60,85,86,87]. An alternative could be the above mentioned multiplexing approaches that would need to be developed, validated, and approved, or NGS provided the reimbursement issues for this technology could be properly solved.

### 2.3. C-Ros Oncogene 1

The *c-ros concogene 1* (*ROS1*) has been identified as a driver mutation of lung cancer cells and primary tumour tissue in 2007 [88]. ROS1 is a member of the insulin receptor family controlling cell cycling and proliferation via several key pathways like *STAT3* and *PI3K/AKT/mTor*. *ROS1* rearrangements have been detected in 2% of AC and like patients with *ALK* rearrangements, most patients are young never-smokers. *ROS1* rearrangements are rarely accompanied by other mutations like *EGFR*, *ALK*, or *KRAS*. The similarity of the clinical profile of patients with *ALK* rearrangement is continued by the response to Crizotinib shown by a phase I clinical trial in which the treatment of *ROS1* positive patients with Crizotinib showed a clear benefit in terms of PFS [89].

### 2.4. PD-L1

Most recently, exactly in October 2015, pembrolizumab was approved for the treatment of *PD-L1* positive NSCLC. *PD1, PD-L1*, and *PD-L2* are the programmed cell protein and its ligands, which play an important role in modulating the T-cell activity. As companion diagnostics, novel antibody assays from Dako, namely the PD-L1_IHC_22C3_phamrDx and the PD-L1_IHC_28-8 antibodies are available, both of which are also FDA proved [90].

### 2.5. Other Biomarkers and Future Aspects

The majority of NSCLC patients harbor oncogenic drivers and several other potential targets, like V-kis-ras2 rat sarcoma viral oncogene homolog (KRAS), the human epidermal growth factor receptor 2 (HER2), v-raf murine sarcoma viral oncogene homolog B1 (BRAF), phosphoinositide-3-kinase caralytic alpha polypeptide (PIK3CA), c-mesenchymal-epithelial transition mitogen (c-MET), activated protein kinase (MAP2K1), fibroblast growths factor receptor (FGFR), discoidin domain receptor 2 (DDR2), phosphatase and tensin homolog (PTEN), the neuroblastoma RAS viral oncogene homolog (NRAS), proteinkinase B (AKT), rearranged during transfection (RET), and many others have been identified for evaluating in targeted therapy. For most of these markers molecular testing methods are already established, such as FISH for HER2, RET, and FGFR, or PCRs for PIK3CA. Pyrosequencing remains a robust and sensitive method for detection of mutations, for which commercially available IVD assays exist and that are commonly used for NRAS, BRAF, as well as for the above mentioned markers EGFR and KRAS [19,20]. However, currently it remains to be clarified how this plethora of biomarkers can be tested on an economically acceptable basis and be reimbursed by the healthcare systems. Novel approaches in NGS sequencing appear to overcome this problem but the analysis is rather time consuming and the method, itself, too expensive for broad usage [49,52,55,91,92,93,94,95,96,97,98,99,100,101,102,103].

It is worth noting that *MET*, the mesenchymal-epithelial transition gene/protein, could be amplified in NSCLC with T790M *EGFR* mutation. This mutation is indicative and causative for TKI resistance, and as a secondary event *MET* amplification could be observed with approved FISH assays [104].

Due to the widespread use of next-generation sequencing (NGS), the next years will generate additional numerous potential targets and the overwhelming success of epigenetics in other malignant disease also promises new molecular options in NSCLC, it will be a challenge to translate each oncogenic driver in a targeted therapy. However, not all will be suitable for clinical application, but molecular-guided therapy is already reality in treatment of lung cancer patients and selected patients will benefit from targeted therapy.

### 2.6. Cost Effectiveness, Facilitation, and Failures of CDx and Multiplexing

A great bias in molecular diagnostics is that, frequently, cost-benefit studies are lacking that would confirm that proper and timely molecular diagnostics may appear expensive in the first line but are suitable to save subsequent budgets required for treatments. Molecular diagnostics could avoid treatment of patients that would not respond to a therapy and, on the other hand, opens the therapy for other patients by identifying those that are eligible of a specific therapy. Unfortunately, convincing comparative cost benefit analyses that focus on the economic aspects of different methods are also missing for NSCLC. As a consequence, it is sometimes difficult to convince patients and physicians that only those molecular diagnostics should be performed that are currently relevant for a therapy decision. As examples, the decision about the TKI therapy will be made on the status of *KRAS*, *BRAF*, and *EGFR* mutations which, in turn, can either be determined by PCR-based methods, pyrosequencing, or NGS. Thereby, PCR and pyrosequencing would deliver the requested result and enable the oncologist to make a clinical decision. A limitation of the PCR would be that it may not detect novel mutations that may affect the therapy outcome, while this could be possible with pyrosequencing. However, NGS delivers the same information plus further data on other regions of the tested genes, may identify even more mutations and combinations of mutations that otherwise are rarely detected due to stepwise laboratory algorithms not testing all in parallel for economic reasons. Anyway, while the first two methods will be reimbursed by most health care systems, NGS will not (yet) generally be reimbursed, although the gain of knowledge would be a benefit for the future. Thus, it should be worth thinking about this investment now to save resources in the future.

NGS would also address the concept of multiplexing analyses, which are frequently used in other disciplines, such as microbiology and virology. Multiplexing would allow the simultaneous analysis of several parameters, which, if sophistically interpreted, would lead to much better therapy decisions in the future. A prerequisite would be that multiplexing is also used in order to further specify the patient groups into molecular phenotypes in a more detailed way than previously performed, as a distinct pattern of parameters may react differently on a given therapy concept.

## 3. Summary and Conclusions

Despite recent progress having been made in the development of CDx required for targeted therapy of lung cancers, the field of research has just passed the first steps on a long road. For routine usage, and broad and cost effective analyses of therapy-relevant mutations and treatment monitoring, the existing methodology is not sufficient. Cheaper methods for whole exome sequencing, or whole-genome sequencing combined with sophisticated and flexible interpretation and analyses tools that rapidly identify and distinguish confirmed therapy relevant mutations, putative therapy relevant mutations, and additional mutations, are needed. Furthermore, it is required that more detailed information on the respective molecular target will be generated which, in turn, will be associated with a clinical therapy response.

Moreover, it should be the foremost goal to define basic terms, such as mutation and polymorphism, that are commonly used in association with CDx methods but are not well defined, although the opposite is suggested by textbooks and stakeholders [105]. If both prerequisites were fulfilled, we would reach the next milestones in personalized and precision medicine.
